# Developing a Visual Attention Assessment for Children at School Entry

**DOI:** 10.3389/fpsyg.2018.02496

**Published:** 2018-12-07

**Authors:** Tanja Prieler, Clare Wood, Jenny M. Thomson

**Affiliations:** ^1^Department of Human Communication Sciences, The University of Sheffield, Sheffield, United Kingdom; ^2^Department of Psychology, Nottingham Trent University, Nottingham, United Kingdom

**Keywords:** visual attention, visual attention assessment, TVA, children at school entry, visual attention development

## Abstract

Whereas young children’s visual attention has been explored in a number of previous studies, so far it has not been investigated by an assessment based on Bundesen’s Theory of Visual Attention (TVA). TVA is a prominent visual attention model that has been widely used as foundation in studies targeting older children, adolescents or adults. In this paper we explore the utility of adopting TVA to explore the visual attention of 4- to 5- year olds and present the development of a simplified adapted version of a TVA-based assessment designed for this age group. Key assessment alterations included the substitution of letter stimuli with black and white symbols and the reduction of assessment duration. The suitability of the assessment for the target age group was subsequently tested in two consecutive studies (Study I: *N* = 43; Study II: *N* = 24). Study results show that measuring visual attention based on a simplified TVA-based assessment appears feasible in such a young age group, provided that the study design takes into account the capabilities of these young children. The authors argue that by adopting this kind of visual attention assessment the relationship between visual attention development and early learning could be better understood.

## Introduction

Although we tend to believe that we are processing all details when looking at an object or watching a scene, human visual systems are only able to focus on a small central area within the visual field, caused by over representation of central vision in the cortex (Horton and Hoyt, 1991). Following Corbetta (1998), selective visual attention can therefore be understood as the mechanism that allows individuals to select important stimuli for further processing while at the same time ignoring other visual inputs. Research has differentiated between overt and covert visual attention. While overt visual attention is relying on the eye fixation on certain stimuli, covert attention includes neural adjustments for paying attention to something without the movement of the eyes (Wu and Remington, 2003). This latter mechanism can for instance be observed when we become aware of motion taking place in the peripheral areas of our vision while being focused on something else. Some actions, such as scanning a text, on the other hand, can only be performed by overt attention and therefore quick eye movements (Findlay and Gilchrist, 2001), so-called *saccades* (Cassin and Solomon, 1990). Nevertheless, covert and overt attention often work in close collaboration: While covert attention enables an individual to perceive a specific area of interest, the following overt saccades enable acquisition of further information (Deubel and Schneider, 1996). Visual attention is also subject to a complex interplay between current goals, selection history and physical salience (Awh et al., 2012).

Research on the development of visual attention has investigated the maturation of different visual attention processes as well as their impact on individuals’ learning and memory development (e.g., Ross-Sheehy et al., 2011; Markant and Amso, 2013; Shimi et al., 2014). These developmental research studies are based on Posner’s Attention Network Theory (ANT). This is a theoretical framework of attention as a whole, defining it as an entity consisting of three independent neural networks (executive, orienting, alerting) (Posner and Petersen, 1990). However, given the existence of more elaborated models of visual attention specifically, our understanding of visual attention development could be more fine-grained. Understanding the development of visual attention today is arguably more important than ever with children’s learning involving increasingly complex visual environments. This is especially true of digitally presented information which commonly features complex, hyperlinked text and non-linear presentation formats.

A particularly prominent visual attention model is Bundesen’s Theory of Visual Attention (TVA) (Bundesen, 1990, 1998). TVA is a mathematical model that defines visual attention as unifying the processes of attentional selection and visual recognition, which are engaged in a parallel processing ‘race.’ That is, caused by the limited storage capacity of visual short term memory (VSTM), objects in the visual field are simultaneously recognized and selected, and compete against each other for representation in the VSTM store (Bundesen, 1990). TVA therefore offers an alternative interpretation to other theories considering these two processes as happening serially (Posner and Rothbart, 2007) and separately, either viewing attentional selection as a requirement for recognition (Broadbent, 1958), or claiming recognition precedes selection (Deutsch and Deutsch, 1963).

According to TVA, an individual’s visual attention profile can further be defined and measured based on five attentional parameters: visual processing speed, storage capacity of VSTM, efficiency of attentional control, visual perception threshold and spatial bias of attention (Bundesen, 1990). In order to quantitatively investigate these parameters, two experimental tasks need to be performed: a whole report task and a partial report task (Duncan et al., 1999). In the whole report, participants are asked to name as many stimuli (simple visual objects, usually letters, typically displayed for < 200 ms) as they can identify on a screen. Similar conditions apply for the partial report, however, participants are asked to only report target stimuli while ignoring distractors (distinguishable through cues such as different colors).

This method of TVA testing was initially designed by Duncan et al. (1999), who investigated patients’ attention after suffering from a stroke. Since the late 1990s it has been widely used and has acted as a theoretical foundation for visual attention studies in different areas of research (Bundesen and Habekost, 2008, 2014; Habekost, 2015). The broad application of TVA-based attention assessments led to Duncan et al.’s (1999) experimental design being altered to either take account of specific needs of a study’s participant group, or to focus on investigating a specific hypothesis related to attention. Variants of TVA-based assessment include exchanging letter stimuli with digits (Starrfelt et al., 2009), short words (Habekost et al., 2014), or faces (Peers et al., 2005). Others studies have changed the format of displaying symbols – e.g., Habekost and Rostrup (2007), who displayed symbols in a circle as opposed to the usual square or line – while Peers et al. (2005) displayed just one stimulus to focus on assessing visual processing speed. In addition, the CombiTVA developed by Vangkilde et al. (2011) was introduced as a second paradigm for TVA-based assessment and has been applied in more recent studies (e.g., Dyrholm et al., 2011; McAvinue et al., 2012; Habekost et al., 2014): in this assessment the whole and the partial report trials are mixed in order to limit the total number of parameter estimates.

Whereas Bundesen’s TVA has therefore been adopted in a number of studies to assess visual attention of target groups with different characteristics, so far no study has implemented a TVA-based assessment to investigate the visual attention skills of young children aged 4–5, who are yet unable to read and write. Instead, studies investigating visual attention in young children have applied either the original or a slightly modified version of a visual attention test developed by Fan et al. (2002), which is based on Posner’s ANT (Posner and Petersen, 1990) (e.g., Mezzacappa, 2004; Rueda et al., 2004; Mullane et al., 2011, 2016). However, it is argued that, apart from ANT not being an explicit visual attention theory, the assessment used in these studies to investigate young children’s visual attention skills has another limitation. That is, ANT assessment comprising a flanker task (Eriksen and Eriksen, 1974) and a spatial cueing task (Posner, 1980), does not directly test an individual’s visual attention *span –* characterized as the maximal string of characters that can be simultaneously processed within a single fixation (Valdois et al., 2004) –, but rather children’s ability to focus on targets while disregarding distractors.

The aim of this study was therefore to test the suitability of an adapted and simplified version of a TVA-based visual attention assessment to investigate children’s visual attention profile at school entry in the United Kingdom when they are between 4–5 years old. By combining a visual attention span task (whole report) and a target detection task (partial report) we argue that a simplified TVA-based assessment can better provide data that specifically targets visual attention in such a young age group. A further key advantage in the practical application of TVA-based assessment, making it especially useful for testing young children, is that both tests (whole report and partial report) are simple in design and relatively straightforward in terms of task demands. Finally, the possibility of adjusting TVA-assessments according to the specific participant group, as well as different theoretical interests, makes it attractive for application in research studies. Visual attention may be an important precursor ability in learning to read and so having an assessment tool that can empirically test this relationship is important. Understanding the development of visual processing is made even more pertinent by the potentially new visual attention challenges and affordances created by digital text that are increasingly confronting emerging readers.

To this end, we were particularly interested in children’s visual attention profile at school entry. Much research points to the developmental status of certain skills at school entry, for example, language skills, and reading-related skills being highly predictive of later school success (Scarborough, 1998; National Early Literacy Panel, 2008). When looking at studies adopting TVA-based visual attention assessment for researching older children or adolescents, from 6 to 16 years, the majority of studies have been conducted in the field of reading research. These studies have investigated the links between visual attention abilities and reading development or reading difficulties (e.g., developmental dyslexia), consistently finding a relationship between these two skills (Prado et al., 2007; Bosse and Valdois, 2009; Dubois et al., 2010; Lobier et al., 2013). Research mainly focuses on an individual’s basic visual efficiency of stimuli processing (usually letters), represented by the parameters of visual short term memory capacity and visual processing speed. They can be further divided into studies applying the original, full TVA assessment as developed by Duncan et al. (1999) (e.g., Dubois et al., 2010; Stenneken et al., 2011; Lobier et al., 2013), and studies that have used an adapted and simplified version of the TVA assessment. The latter was designed to accommodate for the relatively young age of their participants (Valdois et al., 2003; Bosse et al., 2007; Prado et al., 2007; Bosse and Valdois, 2009). One of the main characteristics of the original assessment procedure is its length: In search of best possible estimations, participants tested on Duncan et al.’s (1999) assessment design perform between 252 and 672 trials across 12 different trial types on the whole report, with variations including different display positions and exposure times. The same applies to the partial report where participants are tested on 512–672 trials across 16 different display types (Duncan et al., 1999). In comparison to this, the whole report in a simplified version of TVA assessment – also referred to as visual attention span task (Valdois et al., 2003) – is characterized by a strongly reduced number of trials. The assessment consists of 20 random five-letter strings (based on 10 consonants) displayed in the center of the screen for a fixed time of 200 ms. The partial report in these studies is equally characterized by a very low number of trials, consisting of 50 five-letter strings displayed for 200 ms, identical to those used for the whole report. In addition, participants were asked to report one letter within the string, cued with a vertical bar. This is quite different to the original partial report in which letters were presented in different positions across the screen, with color being used as a cue. While fewer trials give the advantage of a shorter task, it potentially also makes the results less reliable due to a loss in power. In addition, while simplified versions help to overcome challenges associated with testing young children, a further consequence of these simplifications is that there is also no estimation of the five different parameters of visual attention based on TVA (Bundesen, 1990). Instead, a score based on the percentage of letters accurately reported on average in the whole report (storage capacity of VSTM), as well as the percentage of accurately reported targets in the partial report (efficiency of attentional control) is calculated.

Considering children at school entry, it seemed logical to apply a simplified and short version of the TVA-based assessment, similar to the studies discussed above. However, while these previously conducted simplified TVA-based studies have inspired the development of the assessment introduced in this paper, we intended to create a tool that would allow (a) the assessment of young children who are yet unable to read – by exchanging the usually used letter stimuli with simple black and white symbols –, and (b) a more in-depth investigation of storage capacity of VSTM, efficiency of attentional control, as well as spatial bias of attention.

The current paper presents findings of a study conducted to develop a simplified TVA-based visual attention assessment to investigate children’s visual attention skills at school entry taking into account the abilities of relatively unschooled children, whose wider attention capacities are still developing. The proposed study consists of two smaller studies investigating whether the developed assessment differentiates performance between individuals of the target age group (4–5 years old).

The following research questions guided this study:

(i) Is the newly developed visual attention assessment internally reliable?
(ii) Is there evidence of floor or ceiling effects in participants’ performance on the whole report and partial report tasks?
(iii) Does performance on the tasks improve with age?
(iv) What are suitable exposure durations for the whole report task for children aged 4–5?

### Background to Test Design

The study was designed based on the following considerations:

Concerning the number of elements to display within a string for the whole report task, the most common precedent for older populations (Duncan et al., 1999; Bogon et al., 2014) was five symbols (i.e., letters) in each trial of the whole report task. This design decision was itself based on the results of previous studies exploring the question of how many items can be stored in an individual’s visual short term memory (VSTM). Whereas research on adults’ capacity limits has produced more or less converging evidence around the estimate of 3–4 simple elements (Vogel et al., 2001; Astle and Scerif, 2011), studies on the development of VSTM capacity, as well as visual working memory, in infants and children across a wide age range, are more diverse (Ross-Sheehy et al., 2003; Cowan et al., 2005, 2006; Oakes et al., 2006; Riggs et al., 2006, 2011; Simmering, 2012; Isbell et al., 2015). On the one hand, this is due to considerable variability in the amount of visual information retained by individuals of the same age (Conway et al., 2003, 2008). On the other hand, Simmering (2016) pointed out that diverse results are caused by the fact that different study designs are used to capture the limits of VSTM in children, for example, often change preference tasks are used with infants, whereas change detection tasks are used with children and adults. Ross-Sheehy et al. (2003) for instance used a change preference task (Luck and Vogel, 1997) with young infants (6–13 months of age). Based on the study results they concluded that VSTM seems to develop rapidly within the first 12 months of life, when VSTM reaches adult levels of 3–4 items (Ross-Sheehy et al., 2003). However, these findings were highly contested by Cowan et al. (2005, 2006), and Riggs et al. (2006, 2011) who suggested that VSTM continues to develop until the end of later childhood. Finally Isbell et al. (2015), going one step further, suggested that even 16-year old’s capacity of VSTM is still lower than those of tested adults.

Based on these diverging findings and due to the fact that this study’s version of the visual attention task – exchanging letters with black and white symbols of monosyllabic high frequency words – had not been tested before, it was hard to predict how many symbols needed to be displayed for 4- to 5-year olds. For Study I we decided to use a three instead of a five symbol whole report task, displaying symbols in columns on the right and the left side of the screen as presented by Bogon et al. (2014), who researched 9- to 10-year olds. The decision to use a three symbol whole report task in Study I of this study was based on Sørensen and Kyllingsbæk’s (2012) study that looked at preschool children’s VSTM capacity (K). Their results suggested that preschool children’s K is much lower when using a picture based task - compared to a task adopting less complex symbols such as letters or numbers - and was determined to be around 1.90 (*SD* = 0.74). However, it has to be noted, that Sørensen and Kyllingsbæk (2012) again did not use a whole report paradigm (Sperling, 1960) to estimate K, but a change detection paradigm (Phillips, 1974; Pashler, 1988), when assessing children’s visual attention skills.

Another key variable that needed to be determined in order to get a good estimation of visual attention span was range of exposure durations: Regarding the fixation of the maximum exposure durations for each task, the majority of TVA-based assessment studies (e.g., Bosse et al., 2007; Prado et al., 2007; Bosse and Valdois, 2009; Dubois et al., 2010; Lobier et al., 2013; McAvinue et al., 2015) set a maximum exposure duration at 200 ms. While not always discussed, this decision appears to be based on adult eye-tracking studies that examine gaze and saccade behavior; for example, Dubois et al. (2010) stated that their decision to set the maximum exposure duration in their TVA based study design at 200 ms was made in order to avoid saccades. To allow comparison with previous studies, it was decided to adapt Study I in line with the vast majority of previous studies applying TVA-based assessments (Valdois et al., 2003; Bosse et al., 2007; Prado et al., 2007; Bosse and Valdois, 2009; Dubois et al., 2010; Lobier et al., 2013; McAvinue et al., 2015; Van den Boer et al., 2015), and therefore also set the maximum exposure duration at 200 ms.

## Study I

### Materials and Methods

#### Participants

Forty-three Reception Year children (mean age: 4.8, *SD* = 3.6 months; 26 girls, 17 boys) from a United Kingdom primary school participated in the study. All children had received approximately 7 months of instruction at the time of the assessment and had normal or corrected to normal vision. The sample included 5 participants with English as an Additional Language (EAL) and 7 participants with Special Educational Needs (SEN), with no child being categorized as both. No one was excluded from the sample as their performance was not significantly different to the performance of their non-SEN and non-EAL peers. The majority of children had a White British ethnic background (70%), with the other participants belonging to a variety of backgrounds including Black Caribbean, White Eastern European, Chinese, and other mixed backgrounds (30%). 37.2% of all tested participants were eligible for free school meals, used as a proxy for socioeconomic status. Both studies reported in this manuscript were approved by the University of Sheffield (United Kingdom) Ethics committee and written parental informed consent, as well as verbal child assent was obtained.

#### Procedure

All participants were assessed individually in one 20 min long session in a vacant classroom. In order to ensure consistency of test administration between the two researchers, both developed the administration protocol together and practiced it before the start of testing. It was also confirmed that there was no significant difference in participants’ performance between the two researchers administrating the assessment [Study I: *t*(41) = -0.19, *p* = 0.85; Study II: *t*(22) = -0.83, *p* = 0.42].

#### Visual Attention Assessment Measure

**FIGURE 1:**
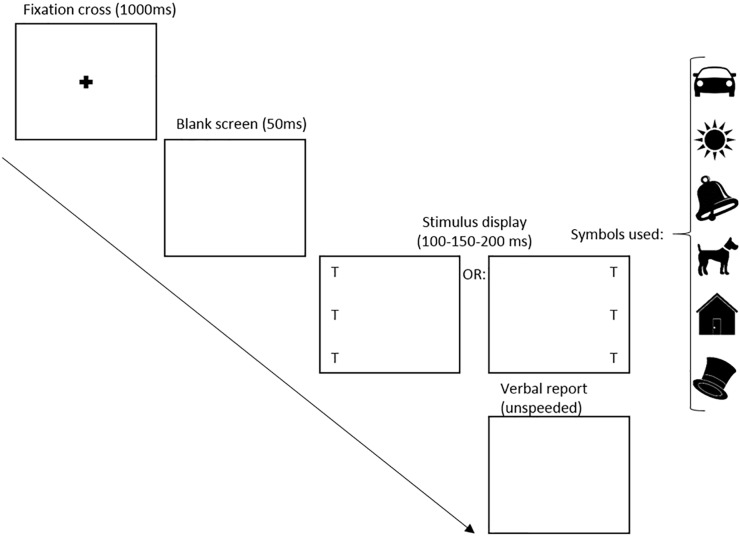
Whole report design Study I. T represents the location of the target images, chosen from the symbols shown on the right side of the figure.

Participants were individually invited to ‘play a game’ on a tablet together with one of the researchers. The assessment consisted of two parts (whole report task and partial report task) the order of administration being counterbalanced across participants. Each task contained an initial practice phase and a main test phase. The test design largely followed the design in Bogon et al.’s (2014) study, investigating the visual attention profile of dyslexic children. This included the parameters of exposure duration, number of symbols displayed, as well as position and arrangement of symbols in both reports. The two visual assessment tasks lasted for about 7–8 min each and were split up into small blocks with a short break in between. In order to make the study accessible for the target group, letter stimuli were replaced by illustrations of familiar objects. These were displayed as black and white symbols to ensure that the objects were clear but at the same time not overly appealing. Using familiar objects followed the design precedent of Sørensen and Kyllingsbæk (2012) and allowed for more control over word length, word frequency and image discriminability compared to other types of symbol such as shapes or colors. Stimuli were presented using the open-source graphical experiment builder Open Sesame on a Samsung Galaxy Tab 4 for Education (10.1 inch 1280 × 800 LCD). The refresh rate was 16.21 ms and symbols were randomly chosen from the following set: car – bell – house – sun – hat – dog (Figure 1). Decisions on which symbols to choose were based on Snodgrass and Vanderwart’s (1980) list of 260 stimuli: From this list, six symbols were chosen depicting nouns having the following criteria: high frequency (>38 occurrences per 1 000 000, Brysbaert and New, 2009), consisting of not more than one syllable, high concept familiarity ratings (Barry et al., 1997), and belonging to different semantic categories: transport – instrument – dwelling – celestial body – clothes – animal.

Before the start of each assessment the child was shown symbols used in the task and was asked to identify each one of them. This way researchers ensured that children were able to name the symbols independently and confidently before the beginning of the task. Each child was then asked to sit at the table and to hold the tablet with both hands, placing their thumbs on thumb markers placed on the right and left bottom corner of the tablet case. In addition, participants were instructed to make sure that the tablet stayed in the area that had been marked on the table with colored tape (15 cm away from the desk end). Before starting the actual testing, children completed four trials in an initial practice phase together with the researcher. During this phase participants were given feedback and were reminded of the task instructions when needed. The main testing was not started before researchers were confident that the child fully understood the task.

##### Whole report task

###### Test administration

Following a centered black fixation cross on a white background (1000 ms) and a subsequent white screen (50 ms), 3 symbols were presented in a vertical column either to the left or to the right of the fixation cross position as shown in Figure 1. Symbols were separated by 2 cm and shown at three different exposure times (100 ms – 150 ms – 200 ms). Symbol strings presented in each trial spanned 10 cm on the display screen, with the symbol sequences being unique in each trial of the study. In line with other studies (Duncan et al., 1999; Bogon et al., 2014), each symbol appeared only once on a given trial. Assuming that the tablet was held at a distance of 30 cm, the symbol string would subtend approximately 18.9°. After each trial of the whole report task participants were asked to make an un-speeded verbal report of as many symbols they were able to recognize on the screen. Analysis showed that performance was similar across symbols. The researcher recorded the symbols in the order they were reported. To allow data checking after the live session, a voice recorder was used to record the answers. Each child completed 42 whole report trials – 7 iterations × 2 positions (left or right) × 3 exposure durations – divided into 4 blocks. After each block (consisting of 10–11 trials), the assessment was briefly paused and the child was given an achievement sticker. Upon completion the child engaged in a short physical activity (action rhyme) to allow for a break before the second half of testing.

###### Data analysis

The whole report task was used to evaluate participants’ performance on how many symbols they were able to name in each trial on average, in relation to the three different exposure durations. This way possible differences between participants’ visual short term memory span could be yielded, defined as the amount of visual elements which can be processed in parallel in a multi-element array during a single fixation (Bosse et al., 2007). According to TVA (Bundesen and Habekost, 2008), a visual object is stored in an individual’s short term memory by encoding of this object’s features into the visual short-term memory store. However, this memory store only has very limited capacity, referred to as parameter *K*. While this study’s whole report design also enables investigation of spatial bias of attention, this data is not reported in this manuscript, given the main aim of the study, i.e., exploring the suitability and feasibility of the test for the target group of 4- to 5-year old participants. Thus, for analyzing the data of the whole report in this study, the number of correctly identified symbols was totaled for each trial and a mean was computed to create a VA score for each participant.

However, since authors of this study argue that VSTM storage capacity K as computed by Bundesen (1998) and Duncan et al. (1999) and the VA span scores (Bosse et al., 2007) – albeit not being completely identical – closely correlate with each other, links were made between this study’s results and TVA-studies’ results concerning VSTM storage capacity (K) when discussing the results of this study.

##### Partial report task

###### Test administration

**FIGURE 2:**
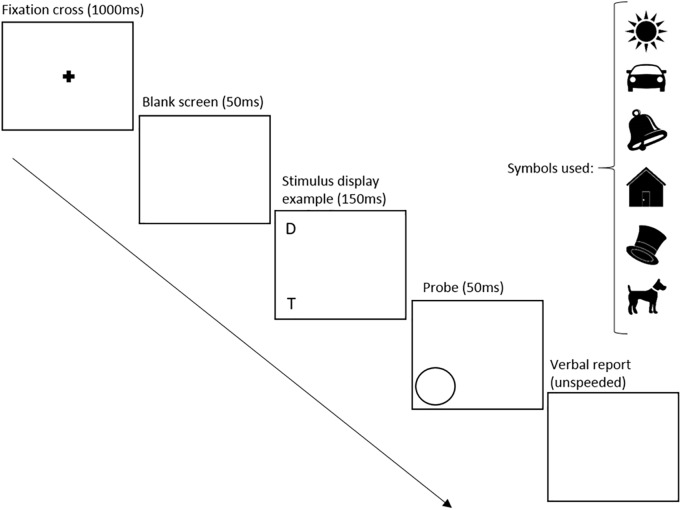
Partial report design Study I and II. T represents the location of the target image, while D represents the location of the distractor image.

**FIGURE 3:**
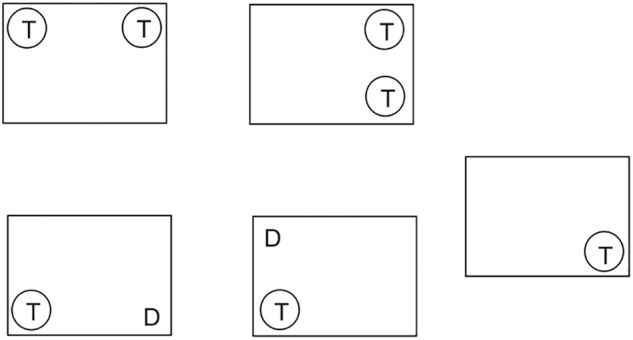
Symbol position in the partial report, Study I and II. T represents the location of the target images, while D represents the location of the distractor images.

This task started similarly to the preceding one, with an explanation of the task followed by four practice trials. At the start of each trial, a central fixation point was presented for 1000 ms followed by a blank screen for 50 ms. In each of the trials there were four array locations (upper left, lower left, upper right, lower right) arranged in a square (12 × 18 cm) around fixation. The array locations are 6 cm apart vertically and 9 cm horizontally. Assuming that the tablet was held at a distance of 30 cm, the rectangle containing the symbols would subtend 19.8° diagonally. In each trial, one or two symbols were displayed for 150 ms at a time and the child was instructed to report the target symbols only. Figure 2 shows that the probe indicating a target was a circle, presented for 50 ms at the location where the target symbols had previously appeared. In different arrays, a target was presented either alone, in pairs, or together with a distractor as illustrated in Figure 3. In addition, targets were presented alone in each of the four locations, while pairs of targets and pairs of targets and distractors were always presented in a row or a column (Duncan et al., 1999). Participants’ unspeeded oral responses were recorded by the researcher who subsequently started the next trial. The partial report was broken into 6 blocks (10 trials each). Similar to the whole report task, the whole procedure was recorded and children received sticker reward in between, as well as by completion of the task.

###### Data analysis

In line with considerations regarding the main aim of the study and the resulting depth of analysis of whole report data, a simplified analysis was also conducted of partial report data, focusing on investigating top–down attentional control. This was achieved by looking at how many targets and how many distractors participants reported on average across all tasks, as well as at correct versus incorrect trials and types of errors made. However, the test design adopted for this study could also be used in future studies to investigate spatial bias of attention.

### Results

#### Is the Adapted Visual Attention Assessment Internally Reliable?

Internal reliability of the adapted visual attention test was evaluated using Cronbach’s alpha, a value that quantifies the extent to which all the trial items in a test measure the same construct (Cronbach, 1951). The Cronbach’s alpha was α = 0.95 for the whole report and α = 0.91 for the partial report task, representing a high level of consistency.

#### Is There Evidence of Floor or Ceiling Effects in Participants’ Performance on the Whole Report and Partial Report Tasks?

##### Whole report results

To examine whether floor or ceiling effects could be identified, overall performance of all participants on the whole report task was examined. The mean number of reported symbols in all trials by all 43 participants was 1.27 (*SD* = 0.81) with a range of 0–3 symbols. When looking at children’s highest score across trials, i.e., their maximum performance, 17 participants (39.5%) achieved a highest score of 1, 8 participants (18.6%) a highest score of 2, and 18 participants (41.8%) a highest score of 3. When looking at symbols not correctly reported, 76.3% were non-responses and 23.7% were an incorrect symbol. No floor or ceiling effects were observed (Table 1).

**Table 1:** Mean, standard deviation (SD), median (MD), and range of the number of reported symbols per trial for whole report task, Study I and II.

	Mean	*SD*	MD	Range
Study I	1.27	0.81	1	0–3
Study II	1.66	0.85	2	0–4

##### Partial report results

Partial report trials varied in terms of both the number of targets and number of distractors within any single trial. As a result, performance could be evaluated in terms of total number of targets reported for an individual *across* trials, as well as according to within-trial performance.

**FIGURE 4:**
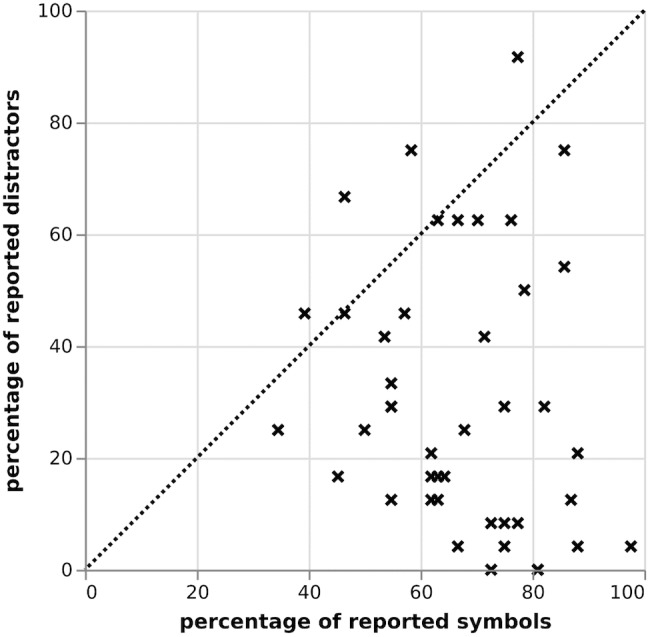
Correlation between reported targets and distractors in % in the partial report, Study I.

Figure 4 reports the percentage of targets/distractors reported across trials for each individual. Across 43 participants, 67.2% of the target symbols were reported correctly. Distractor symbols were reported 30.4% of the time. It was further revealed that whereas 30 participants (69.8%) showed high abilities to report targets, while disregarding distractors (participants well below the line in Figure 4), 13 (30.2%) participants reported targets and distractors indiscriminately and with roughly the same probability (participants close to the line in Figure 4).

Analyzing the data on a by-trial basis, across all 43 participants, 52.3% of all trials were reported correctly, 13.9% were reported partially correct – one out of two targets displayed – and the remaining 33.8% were reported incorrectly. Of those error reports, 46.0% were false alarms (i.e., a symbol was given that was not a target or distractor), 35.0% reported a distractor instead of target, 14.1% were no responses, and 4.9% were multiple errors – i.e., a report of a distractor and an additional wrong symbol (see Table 2). The correlation between correct trials and trials with reported distractors was significant (*r =* -0.62; *p <* 0.001). The correlations between correct trials and the remaining error types were all non-significant.

**Table 2:** Mean, standard deviation (SD), median (MD) and range of partial report responses in Study I in per cent (number of trials = 60).

	Mean	*SD*	MD	Range
Correctly reported trials	52.3%	16.8%	51.7%	23.3–95.0%
Partially correct responses	13.9%	14.7%	8.3%	0.0–40.0%
Error responses:	33.8%	7.2%	6.7%	0.0–40%
	Distractor responses	36.0%		
	False responses	44.4%		
	No responses	14.6%		
	Multiple error responses	5.0%		

#### Does Performance on the Tasks Improve With Age?

**FIGURE 5:**
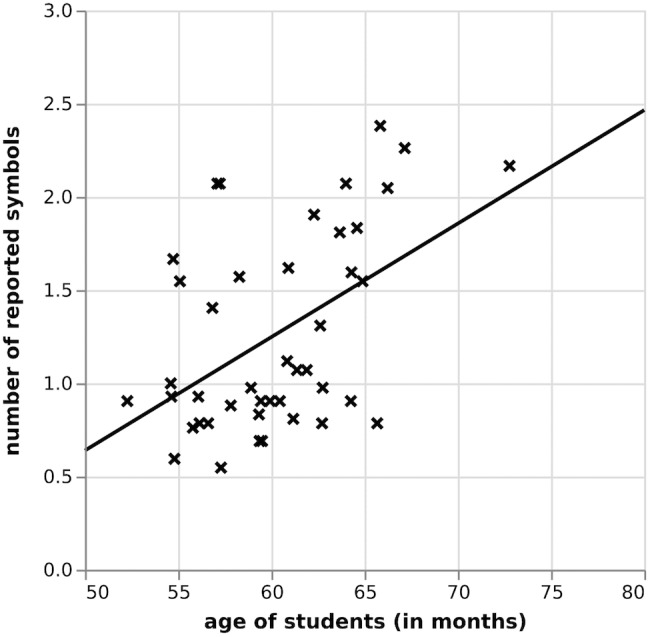
Correlation between number of reported symbols and pupils’ age in the whole report, Study I.

To explore developmental effects, first, whole report results were used to examine the correlation between numbers of reported symbols and age of participants in months, followed by an examination of a potential age effect in the partial report results. Figure 5 shows that there was a significant relationship (*r* = 0.37; *p* = 0.02) between the performance of participants in the whole report task and their age in months. On average, the older participants were, the higher the number of symbols they were able to report. In the partial report, the relationship between age of students and percentage of correctly reported trials was also significant, *r* = 0.45, *p* < 0.01.

#### What Are Suitable Exposure Durations for the Whole Report Task for Children Aged 4–5?

Whole report data showed a slight increase of reported symbols with an increase in exposure duration, with the mean number of reported symbols at 100 ms being 1.23 (*SD* = 0.52), at 150 ms being 1.26 (*SD* = 0.55), and at 200 ms being 1.30 (*SD* = 0.58). There was no significant difference between the number of reported symbols per exposure duration [*F*(3,43) = 0.14, *p* = 0.87]. These findings suggest that the selected exposure duration times for Study I (100 ms – 150 ms – 200 ms) only seemed to have a small impact on the symbols participants were able to report in the whole report task.

### Summary of Study I

The purpose of this first study was to test a newly developed visual attention assessment for children in the first year of schooling – based on Bundesen’s theory of visual attention (TVA) (Bundesen, 1990, 1998).

Based on Study I results, the visual attention test appeared to be internally reliable, showing a high level of consistency in the whole and the partial report.

When looking at the data from the whole report no floor or ceiling effects were observed. The newly developed visual attention test provided a wide range of performance across all 43 participants of the study, suggesting the suitability and sensitivity of the assessment. In line with this, results of the partial report data suggest that the demands of the partial report task – reporting targets while ignoring distractors – could be met by the participants. The assessment captured a range of performance, from participants showing high abilities to name targets while ignoring distractors, to others who had a lot more difficulty with this task.

As with other studies (e.g., Riggs et al., 2011), we have potentially observed a developmental progression from the whole report data analysis suggesting that age of participants had an impact on average number of reported items. This could not be found in the results from the partial report. In contrast to studies with older populations (Habekost, 2015), however, there was a minimal effect on performance as a function of exposure time.

As a final point, while the above results supported the overall utility of the visual attention assessment for the target age group, the researchers noticed that more than a third of the participants struggled with the vertical orientation of the stimuli; as per Bogon et al. (2014) symbols were displayed in a column on either the left or the right side of the screen in the whole report. While some children expressed surprise, stating that they never knew where the symbols would pop up on the screen, others showed mild levels of frustration, blaming the lack of predictability of target locations for failed attempts to recall one or more symbols.

### Implications for Study II

Following the results of Study I, the following changes were made to further improve the suitability of the visual attention assessment for the target group of the study: firstly, since children were challenged by the display of the symbols, the revised version presented the symbols in a horizontal string at the center of the screen. The change in displaying the symbols in a line was further based on the argument that this presentation style was more reflective of the way text is usually presented, i.e., in horizontal lines.

Secondly, the number of symbols shown in one trial was increased to four instead of three. While no overt ceiling effects were observed, given that a significant proportion of children could report all three symbols presented, trialing the use of four symbols was seen as a way to potentially capture greater between-child performance variation.

Further, to improve the sensitivity of the measure to capture performance differences as a result of varied exposure time, the exposure durations were spread out more widely: 70ms – 100ms – 150ms – 200ms – 250ms. It was hoped that by testing five instead of three exposure times, and by increasing the overall time span from 100 to 180 ms, data would be more informative regarding the relationship between exposure time and number of reported symbols.

Since a bigger question remained about the impact upon validity of a reduced number of whole report trials in Study I – 42 trials instead of > 150 in previously conducted TVA studies (e.g., Bogon et al., 2014) – Study II looked into effects of increasing the number of whole report task trials from 42 to 100.

## Study II

### Materials and Methods

#### Participants

Twenty-four Reception Year children (mean age: 5.3, *SD* = 3.1 months; 11 girls, 13 boys) from a United Kingdom primary school participated in this study. All participants had received approximately 10 months of instruction at the time of the assessment. All children had normal or corrected to normal vision and the sample included 1 SEN child. In line with Study I, no child was excluded from testing. All 24 tested children had White British ethnic background, with one out of all tested participants being eligible for free school meals.

#### Procedure

The visual attention test was administered in 20 min long sessions. In line with Study I, all children were assessed individually in an empty classroom by one of the two researchers, using the same administration protocol.

#### Visual Attention Assessment Measure

For the visual attention assessment measure in Study II the assessment procedure was nearly identical with the one in Study I. For the purpose of Study II, however, participants were split into two groups of 12 participants each, with group A performing the revised version of the assessment consisting of whole report and partial report task, and group B performing a longer version of the revised whole report task only.

Whereas the whole report was changed for both groups based on the results in Study I, the same partial report was used for the testing. For group A the two tasks (i.e., partial and whole report) lasted for about 7–8 min each, were split up into small blocks and separated by a break in between. For group B the whole report task lasted for 15 min in total, also split into blocks with in between breaks. In line with Study I, for both groups stimuli were presented on a Samsung Galaxy Tab 4 for Education (10.1 inch 1280 × 800 LCD) and were randomly chosen from the following set: car – bell – house – sun – hat – dog – clock – pot. Based on the increased number of displayed symbols in this study, two symbols (clock, pot) were added to the list of symbols used in Study I, having been chosen according to the same characteristics as the other six (for more details see description of Study I). All other aspects of tablet positioning and practice trial administration followed the procedures outlined for Study I.

##### Whole report task

###### Test administration

**FIGURE 6:**
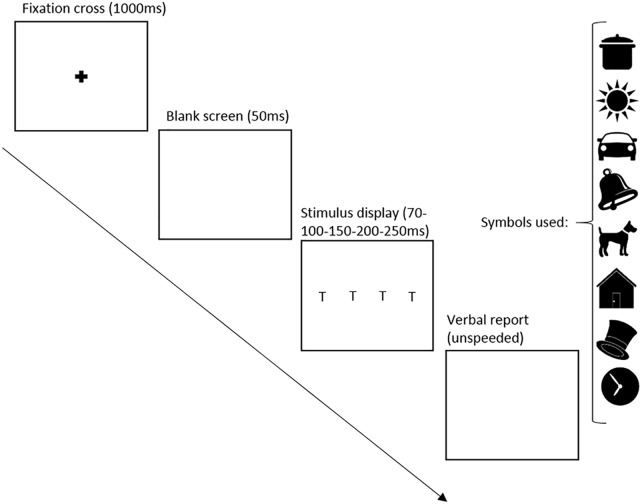
Whole report design Study II. T represents the location of the target images, chosen from the symbols shown on the right side of the figure.

Following a centered black fixation cross on a white background (1000 ms) and a white screen (50 ms), the silhouette of four symbols were presented in a horizontal line in the center of the screen as can be seen in Figure 6. Symbols were shown at five different exposure times (70ms – 100ms – 150ms – 200ms – 250ms). With the string of symbols spanning 18 cm on the screen, symbols were separated by 3 cm each. Symbol sequences were unique in each trial of the study and, following the TVA test design (e.g., Duncan et al., 1999; Bogon et al., 2014), appeared only once on a given trial. With the tablet held at a distance of 30 cm, a symbol would subtend about 33.4°. After each trial of the whole report task the child was asked to make an unspeeded verbal report of as many symbols as he/she was able to recognize on the screen. The researcher recorded the symbols in the order the participant reported them and in addition used a voice recorder throughout the sessions.

Each child of group A completed 40 whole report trials – 8 iterations × 1 position (centered) × 5 exposure durations – divided into 4 blocks. After each block (consisting of 10 trials), the child received an achievement sticker. Participants of group B performed on 100 whole report trials – 20 iterations × 1 position (centered) × 5 exposure durations – divided into 10 blocks of 10 trials, each shortly paused by a reward receiving activity. Whereas participants of group A engaged in a short physical exercise after completing the whole report, group B engaged in physical activities after completing half of the whole report trials.

###### Data analysis

In line with Study I, data from the whole report was yielded to compute individuals’ visual attention spans on the adapted whole report task. In comparing results with those from Study I, the main focus was to investigate whether changes in the study design resulted in stronger correlation between participants’ number of reported symbols and exposure durations and therefore go in line with results from other studies applying TVA (e.g., Duncan et al., 1999; Dubois et al., 2010; Bogon et al., 2014).

### Partial Report Task

The conditions of the partial report task remained unchanged from Study I (see Figures 2, 3) and were performed by children assigned to group A.

### Results

#### Is the Adapted Visual Attention Assessment Internally Reliable?

In line with the results in Study I, internal reliability of the adapted visual attention test was evaluated using Cronbach’s alpha. The visual attention test applied in Study II showed high internal reliability for the results of both groups, with α = 0.93 for the whole report and α = 0.89 for the partial report of group A and α = 0.97 for the whole report of group B.

#### Is There Evidence of Floor or Ceiling Effects in Participants’ Performance on the Whole Report and Partial Report Tasks?

##### Whole report results

To examine whether floor or ceiling effects could be identified, overall performance of all participants (group A and B) on the whole report task in Study II was examined. The mean of number of reported symbols in all trials by all 24 participants was 1.66 (*SD* = 0.85) with a range of 0–4 symbols. Results revealed a wide range between performance of tested children, stretching from 2 participants (8.33%) reporting a maximum of 1 symbol across all whole report trials, the majority of participants (17, 70.8%) reporting a maximum of 2 or 3 symbols across all trials, to 5 participants (20.8%) reporting up to 4 symbols throughout all whole report trials. While results from Study I and II are not directly comparable as the display of symbols in the WR was altered between studies, it is worth noting that performance on the WR in Study II was significantly better than on the WR in Study I [*t*(65) = 3.21, *p* = 0.002]: whereas a significant number of participants in Study I only reported up to one symbol (39.5%) across all whole report trials, nearly all participants (91.6%) in Study II reported up to 2 or more symbols across all trials (Table 1).

##### Partial report results

**FIGURE 7:**
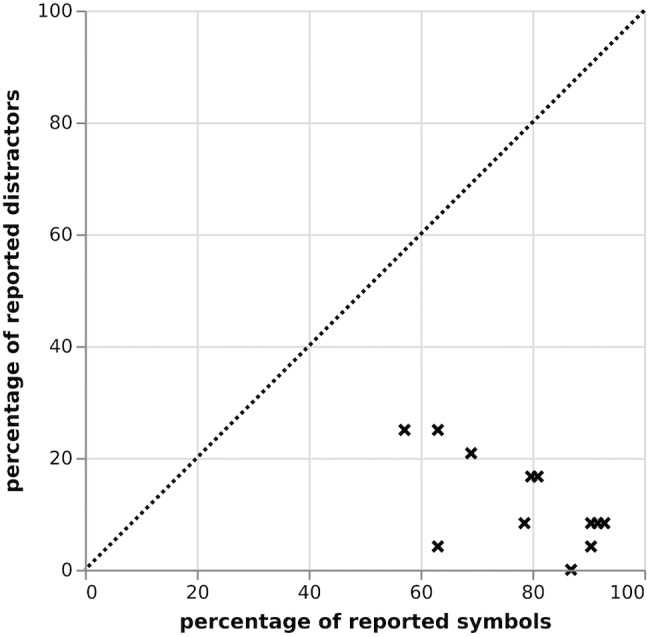
Correlation between reported targets and distractors in % in the partial report, Study II.

As shown in Figure 7, there was a range in performance of all tested participants, yet general performance was relatively strong: when analyzing the percentage of targets/distractors reported across trials for each individual, 78.7% of the target symbols were reported correctly, while distractor symbols were reported only 12.2% of the time. Individuals’ performances on the partial report further showed a significant relationship (*r* = -0.64; *p* = 0.034) between participants’ reported targets (T) and distractors (D), supporting findings that participants tested on the partial report show high abilities to report T while disregarding D (right bottom corner).

Analyzing the data on a by-trial basis, across all 12 participants, 57.5% of all trials were reported correctly, 24.9% were reported partially correct – one out of two targets displayed – and the remaining 17.6% were reported incorrectly. Of those error reports, 33.9% were false alarms, 27.6% reported a distractor instead of the target, 36.3% were no responses, and 2.2% were multiple errors – i.e., a report of a distractor and an additional wrong symbol (see Table 3). The correlation between correct trials and trials with reported distractors was significant (*r* = -0.77; *p <* 0.01). The correlations between correct trials and the remaining error types were all non-significant.

**Table 3:** Mean, standard deviation (SD), median (MD) and range of partial report responses in Study II in per cent (number of trials = 60).

	Mean	*SD*	MD	Range
Correctly reported trials	57.5%	16.8%	60.8%	15.0–71.7%
Partially correct responses	24.9%	9.4%	21.7%	15.0–45.0%
Error responses:	17.6%	6.6%	13.3%	0.0–11.7%
	Distractor responses	27.6%		
	False responses	33.9%		
	No responses	36.3%		
	Multiple error responses	2.2%		

#### Does Performance on the Tasks Improve With Age?

**FIGURE 8:**
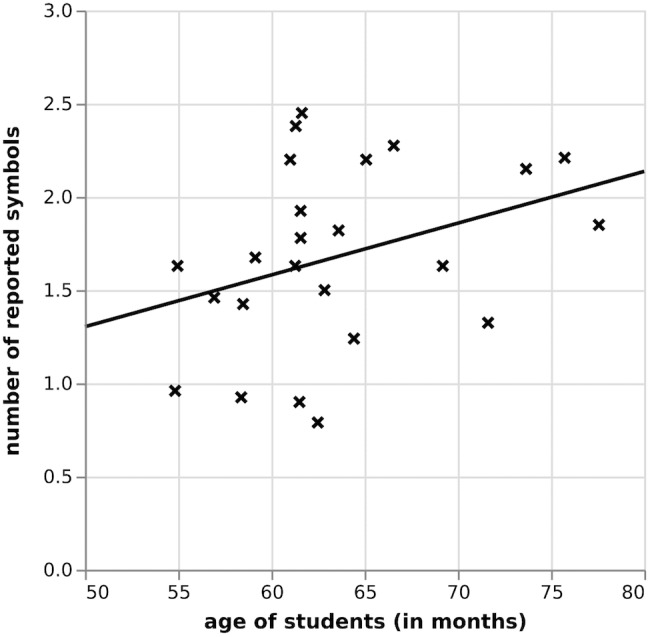
Correlation between number of reported symbols and pupils’ age in the whole report, Study II.

To explore developmental effects, the relationship between number of reported symbols in the whole report task and participants’ age for both groups, was investigated, as can be seen in Figure 8. For this group (*n* = 24) there was no significant relationship (*r* = 0.31; *p* = 0.14) between the performance of participants in the whole report task and their age. Similar results were yielded for performance on the partial report: the correlation between age of student and percentage of correctly reported trials was not significant, *r* = 0.41, *p* = 0.19.

#### What Are Suitable Exposure Durations for the Whole Report Task for Children Aged 4–5?

Whole report data showed an increase of reported symbols with an increase in exposure duration, with the mean number of reported symbols at 70ms being 1.56 (*SD* = 0.58), 100 ms being 1.61 (*SD* = 0.53), at 150 ms being 1.67 (*SD* = 0.46), at 200 ms being 1.77 (*SD* = 0.55), and at 250 ms being 1.79 (*SD* = 0.51). However, there was no significant difference between the number of reported symbols per exposure duration (*F*(5,24) = 0.85, *p* = 0.49).

### Summary of Study II

The purpose of this second study was to test whether the amended version of the visual attention assessment improved its suitability and sensitivity in comparison to the first version, trialed in Study I.

Based on Study II results the adapted visual attention test appeared to be internally reliable, showing a high level of consistency in the whole and the partial report. The similarly high internal reliability for both groups A and B, i.e., children who carried out 40 or 100 trials respectively, suggested that there was not a significant decrement to internal reliability for this age group in carrying out the task with a reduced number of trials.

The data from the whole report showed a wide spread of performance and no evidence of floor or ceiling effects, with the addition of a further symbol to recall providing further differentiation of performance. Equally, the positive correlation between reported targets and distractors in all partial report trials showed that participants tested could perform the task relatively well, yet still across a range of abilities. Regarding the relationship between number of reported symbols and age, while a positive correlation (*r* = 0.31) existed between the age of participants and average number of reported trials (whole report) of a magnitude similar to Study I (*r* = 0.37), the correlation was not significant. This may be due to the low sample size of Study II (*N* = 24) in comparison to Study I (*N* = 43). Contrary to findings from Study I, no age affect was found in the partial report in Study II.

The investigation of the suitability of the exposure durations for children aged 4–5 explored the relationship between the number of reported items and exposure time in the whole report task in Study II. Whereas five instead of three exposure times were used in Study II and the mean of reported symbols per trials (*M* = 1.66; *SD* = 0.85) was higher compared to Study I (*M* = 1.27; *SD* = 0.81), there was no significant difference in performance as a function of exposure duration in Study II. The effect of exposure duration on the number of correctly reported items therefore appears to be lower than in other studies applying TVA with older populations (8 years and older) (Dubois et al., 2010; Lobier et al., 2013).

## General Discussion

The primary purpose of this study was to investigate the accessibility, suitability, and sensitivity of a newly adapted version of visual attention assessment for children at school entry.

As observed in the discussions of Study I and II, the current assessment demonstrated good internal consistency, showed no evidence of floor or ceiling effects and thus captured a range of performance within this age group. These findings suggest that while there was a reduction of trial numbers in order to accommodate for the target age group of this study, the assessment still has enough power to demonstrate reliability. Whole report data revealed that the mean visual attention span across all tested participants lay between 1 and 2 items. Comparing these results to previous studies, no study has reported visual span performance on this specific age group. With slightly older children, Bosse and Valdois (2009) used a simplified TVA-based assessment with 157 6- to 7-year olds. Using a different reporting metric, the researchers looked at the percentage of trials in which all 5 letter strings were correctly reported – which was a mean of 7.3% for their sample population. Calculating a similar metric for the data here, the parallel result would be 1.04% – which is considerably lower. Part of this performance discrepancy may be due to the age difference, and the school transition period representing a time of rapid developmental change in skills relevant to formal instruction. Another factor to consider is that the processing of pictorial symbols, while circumventing the need for letter knowledge, may have a differential processing load to letters. A challenge in creating developmental assessments is the inherent dynamism of the skills being assessed. When creating a measure that is sensitive to a specific developmental window (in this case, emerging readers), by its very nature, the measure may not be as sensitive to, or comparable with, a different developmental phase.

Regarding within-cohort age effects, however, for both the whole report and partial report data in Study 1 there was a significant and positive correlation between age and performance. Finally, in relation to our fourth research question, whereas a positive relationship between an increase in the selected exposure durations and the number of symbols participants reported was observed, the effect was not significant. It will also be valuable to administer this assessment alongside a wider collection of visual processing and attention tests, to fully ascertain the accompanying validity of the measure. Another important aspect of validation will be delineating, as far as possible, the relative contribution of symbol-to-spoken word processing to task performance. Complete separation of visual and verbal processing is arguably not possible within behavioral assessment tasks, but by analyzing test performance controlling for verbal ability, the relative contribution of these skills could be explored.

In the remaining part of this General Discussion, we will further discuss the role of age and exposure duration in relation to measuring visual attention in young children.

### Role of Exposure Duration

An unexpected finding in both Study I and Study II was the lack of significant effect for exposure duration on children’s performance in the whole report task. In Study I, three different exposure durations were employed, of comparable length to studies with adults (100–150–200 ms), whilst in Study II the number and range of durations was extended further (70–100–150–200–250 ms).

In both studies, the mean number of reported symbols did consistently increase as exposure duration increased, thus one explanation for this result is a lack of statistical power. Having reduced the number of overall trials to make the assessment more appropriate to young children, this means that further dividing results as a function of exposure duration may have made it difficult to discern statistically significant effects in the data.

Alternatively, knowing that children process information more slowly than adults (Kail, 1991; Riggs et al., 2006), it could be suggested that studies of visual processing that have relied on adult models of gaze and saccade patterns may not be applicable to young children, especially those with minimal school experience (and thus reduced experience with processing strings of text and symbols). While small-scale studies of children’s visual processing have been reported (e.g., Yang et al., 2002), these findings suggest the need for increased empirical research regarding childhood visual cognition in order to better understand the role of exposure duration in young children’s visual processing.

### Role of Age

To investigate within sample age effects we first focused on the whole report, where we had single performance scores. Regarding the observed relationship between age of participant and storage capacity, the whole report results of Study I showed evidence for cross-sectionally observed developmental differences. This goes in line with other reported findings of visual short term memory capacity continuing to develop during early childhood (e.g., Riggs et al., 2011). At the same time, both significant (Study I) and non-significant (Study II) age effects were found in children’s performance on the partial report.

Another opportunity to consider age effects arose indirectly in relation to the partial report task used in Study I and Study II. The partial report task parameters remained unchanged between Study I and II, and given the sequential order of the studies, the mean age of the children in Study I and II differed by approximately 4 months (mean age of 4.8 years in Study I, mean age of 5.3years in Study II). As reported, the cohort in Study II (who were a different group of children to those in Study I) performed noticeably better, as a group, than those in Study I, with superior ability to ignore distractors. These findings are slightly more difficult to interpret than the within-in study age effects reported for the whole report, as between Studies I and II, the children not only differed in age, but also in the amount of formal instruction they had received at school. The children in these studies were in their first year of schooling and acquisition of skills such as letter knowledge have been shown to have a specific and measurable effect on early cortical visual processing (e.g., Maurer et al., 2005), that is separable to developmental maturation *per se*. This study was not designed to differentiate the specific effect of formal instruction versus age on the development of visual attention, but we note that differing instructional environments, as well as varied international practices when reading instruction commences, may have an impact on visual attention development, and thus the sensitivity of assessment measures across contexts.

### Conclusion

Overall, this study is the first of its kind, to the authors’ knowledge, to show that measuring visual attention based on TVA appears feasible in such a young age group. By adopting this kind of visual attention assessment the role of visual attention in early learning, and the reciprocal relationship between children and their instructional environment may be better understood.

## Ethics Statement

This study was carried out in accordance with the recommendations of the University of Sheffield Ethics Policy Governing Research Involving Human Participants, Personal Data and Human Tissue with written informed consent from all subjects. All subjects gave written informed consent in accordance with the Declaration of Helsinki. The protocol was approved by the University of Sheffield Ethics Administration.

## Author Contributions

TP, CW, and JT designed the study and wrote the manuscript. TP collected and analyzed the data.

## Conflict of Interest Statement

The authors declare that the research was conducted in the absence of any commercial or financial relationships that could be construed as a potential conflict of interest.
